# Non-adherence to guideline recommendations for insulins: a qualitative study amongst primary care practitioners

**DOI:** 10.1186/s12875-022-01760-5

**Published:** 2022-06-13

**Authors:** M. Dankers, M. J. E. van den Berk-Bulsink, M. van Dalfsen-Slingerland, H.J.M.G. Nelissen-Vrancken, A. K. Mantel-Teeuwisse, L. van Dijk

**Affiliations:** 1 Dutch Institute for Rational Use of Medicine, Churchilllaan 11, 3527 GV Utrecht, the Netherlands; 2 Dept. of PharmacoTherapy, -Epidemiology & -Economics (PTEE), Faculty of Science and Engineering, Groningen Research Institute of Pharmacy, University of Groningen, A. Deusinglaan 1, 9713 AV Groningen, the Netherlands; 3 Division of Pharmacoepidemiology & Clinical Pharmacology, Utrecht Institute for Pharmaceutical Sciences (UIPS), Utrecht University, Universiteitsweg 99, Utrecht, 3584 CG the Netherlands; 4 Netherlands Institute for Health Services Research, Otterstraat 118, 3513 CR Utrecht, the Netherlands

**Keywords:** Primary care, Diabetes, Insulin, Guideline adherence

## Abstract

**Background:**

Guideline adherence is generally high in Dutch general practices. However, the prescription of insulins to type 2 diabetes mellitus patients is often not in line with the guideline, which recommends NPH insulin as first choice and discourages newer insulins. This qualitative study aimed to identify the reasons why primary care healthcare professionals prescribe insulins that are not recommended in guidelines.

**Methods:**

Digital focus groups with primary care practitioners were organised. A topic list was developed, based on reasons for preferred insulins obtained from literature and a priori expert discussions. The discussions were video and audio-recorded, transcribed verbatim and coded with a combination of inductive and deductive codes. Codes were categorized into an existing knowledge, attitudes and behaviour model for guideline non-adherence.

**Results:**

Four focus groups with eleven general practitioners, twelve practice nurses, six pharmacists, four diabetes nurses and two nurse practitioners were organised. The prescription of non-recommended insulins was largely driven by argumentation in the domain of attitudes. Lack of agreement with the guideline was the most prominent category. Most of those perspectives did not reflect disagreement with the guideline recommendations in general, but were about advantages of non-recommended insulins, which led, according to the healthcare professionals, to better applicability of those insulins to specific patients. The belief that guideline-recommended insulins were less effective, positive experience with other insulins and marketing from pharmaceutical companies were also identified as attitude-related barriers to prescribe guideline-recommended insulins. One additional category in the domain of attitudes was identified, namely the lack of uniformity in policy between healthcare professionals in the same practice. Only a small number of external barriers were identified, focusing on patient characteristics that prevented the use of recommended insulins, the availability of contradictory guidelines and other, mostly secondary care, healthcare providers initiating non-recommended insulins. No knowledge-related barriers were identified.

**Conclusions:**

The prescription of non-recommended insulins in primary care is mostly driven by lack of agreement with the guideline recommendations and different interpretation of evidence. These insights can be used for the development of interventions to stimulate primary care practitioners to prescribe guideline-recommended insulins.

## Background

Substantial evidence exists that adherence to clinical practice guidelines positively affects the quality of primary care. Guideline adherence has been associated with more patient satisfaction with their treatment [1] and improved patient outcomes [2]. In addition, guideline adherence can improve the process and structure of care [3] and reduce costs [4].

A country with a long history of developing and implementing clinical guidelines in primary care is the Netherlands [2]. National guidelines covering the majority of conditions and diseases in general practice are developed by the Dutch College of General Practitioners (NHG) [5]. Virtually all (97%) Dutch general practitioners have a positive attitude towards those guidelines [6]. Moreover, 89% of Dutch general practitioners believe that guideline adherence contributes to better quality of care. Adherence to these guidelines among general practitioners is therefore generally high, around 75% [6, 7], but varies among types of diseases and recommendations, with some areas of poor adherence [7, 8].

One area with poor guideline adherence is the prescription of insulins for type 2 diabetes mellitus (T2DM) patients. In Dutch general practice, where the majority of insulins for T2DM are prescribed [9], less than 20% of T2DM patients needing insulin treatment starts with the guideline-recommended NPH insulin. Instead, insulin glargine 100 U/ml and insulin detemir – which are mentioned as less favourable, alternative options—are often initiated. In addition, approximately 25% of all insulin users uses one of the newer agents insulin glargine 300 U/ml or insulin degludec, which gained market access in 2013 and 2015, respectively [10]. Those two newer insulins are not recommended, because of the lack of evidence-based advantages in terms of efficacy or safety, and higher costs [11]. The Dutch guideline is in line with most international guidelines for the treatment of T2DM that also favour NPH insulin for insulin-naïve patients and do not recommend the use of insulin glargine 300 U/ml and insulin degludec [12, 13]. In spite of this, the declining popularity of NPH insulin and rapid adoption of newer insulins is a worldwide trend [10, 14–20], resulting in substantial increases in total insulin expenditures [20–24]. Although previous observational research showed that the prescription of newer insulins was related to several patient and practice characteristics, most reasons for this guideline non-adherence could not be elucidated [10]. According to Cabana et al., potential barriers to guideline adherence can be organised in a knowledge, attitudes, behaviour framework, which states that before a guideline can affect patient outcomes, it first affects healthcare professionals’ knowledge, then attitudes and finally behaviour. In this model, the behaviour of the healthcare professional is determined by knowledge (is the healthcare professional familiar with the guideline), attitude (is he or she willing to perform the recommendation) and external barriers (do factors which are beyond their control hamper the execution of the recommendation) [25]. It is yet unknown to what extent these barriers to physician adherence to guidelines also apply for the prescription of guideline-recommended insulins.

To ensure quality of primary care and prevent increasing expenditure on insulins for T2DM patients, insight in the reasons for guideline non-adherence concerning the prescription of insulins is of crucial importance. The aim of this qualitative study is therefore to identify the reasons why primary care healthcare professionals prefer non-recommended insulins, focusing on the prescription of other insulins than NPH insulin for insulin-naïve patients and the prescription of newer insulins to both insulin-naïve and prevalent insulin users.

## Methods

Focus group discussions were performed with primary care healthcare professionals to study their preferences and accompanying argumentation for insulin treatment in T2DM patients. Focus groups were preferred over individual interviews since they allow participants to interact with each other. Focus groups have therefore been associated with a wider range of views and ideas than can be collected by using individual interviews [26, 27].

### Setting

This study was carried out among Dutch general practitioners, practice nurses, diabetes nurses, nurse practitioners and pharmacists. In the Dutch healthcare system, most T2DM patients are treated in primary care [9]. The majority of general practices deploy nurses (i.e. practice nurses, diabetes nurses or nurse practitioners) to take care of T2DM patients [28, 29]. While diabetes nurses and nurse practitioners have a formal prescriptive authority, practice nurses do not [30]. Practice nurses have, however, a prominent role in the management of T2DM patients, including advising general practitioners about the preferred treatment [31]. Pharmacists have no prescriptive authority, but do have an important advisory role in the pharmaceutical treatment in the Netherlands. They were also involved because of their insight in the actual prescription patterns, both from general practitioners and secondary care providers.

Two out of four focus group meetings were held during PharmacoTherapy Audit Meetings (PTAMs). Since this study was carried out during the second wave of COVID-19, recruiting general practitioners outside regular activities would have been extremely difficult. We therefore planned to organise the focus group discussions during regular meetings with general practitioners, so no additional time-investment was necessary. PTAMs are regular meetings between general practitioners and pharmacists (and sometimes nurses) in the same region. PTAMS are organised to exchange information and views about pharmacotherapy with the aim of improving the prescribing and dispensing of medicines [32]. Almost all Dutch general practitioners and pharmacists participate in a PTAM in their region.

Since nurses are not always invited at PTAMs, two additional focus group meetings with practice and diabetes nurses were organised. Since the daily work of these professionals was less bothered by the COVID-19 pandemic, and to obtain a more heterogeneous representation than from PTAMs, these focus groups were specifically organised for the purpose of this research. To attract a broad range of nurses, individual participants were recruited through open enrolment.

### Subjects

Both PTAMs and individual practice and diabetes nurses were recruited by an open call for participation through the newsletter and social media of the Dutch Institute for the Rational Use of Medicine (IRUM). A snowballing technique was used with the participants (PTAM or nurse) being asked to invite other PTAMs or nurses.

The two open enrolment groups with practice and diabetes nurses were organised with at least five participants and a maximum of eight. The number of participants in the PTAMs depended on local situations. All participants gave written informed consent before the start of the focus group discussions. According to Dutch legislation, approval by a medical ethics committee was not necessary, since no patients were involved in this study and the participants of the focus group discussions were not exposed to interventions [33].

### Data collection

We prepared a topic list based on the model of Cabana et al. [25] and argumentation for preferred insulins obtained from literature and a priori expert discussions. The topic list was fine-tuned during several sessions within the research team. Covered topics were the preferred initial insulins, the prescription of newer insulins and corresponding argumentation in the domains of knowledge, attitudes and external barriers.

The focus group discussions were organised in October and November 2020. Due to the COVID-19 pandemic, we used a virtual focus group methodology using Zoom Video Communications. The discussions lasted 45 – 75 min and were facilitated by MD as moderator. MvdB and (in three of four groups) MvD were observers.

### Data analysis

**Fig. 1:**
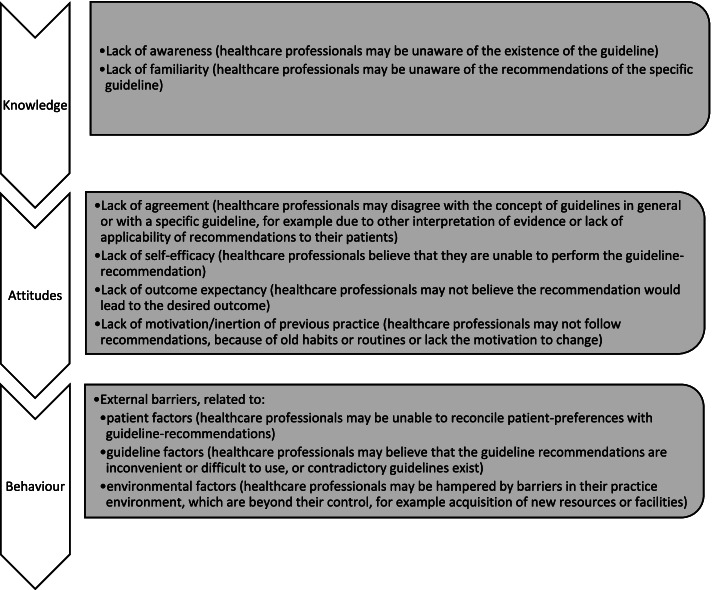
Domains and categories of guideline non-adherence, according to Cabana et al. [25]

The discussions were video and audio-recorded and transcribed verbatim using automatic generated transcripts performed by AmberScript, which were manually verified and corrected. The transcripts were coded and analysed in Atlas.ti 9.1.5.0. Coding was performed with a combination of deductive and inductive codes. Deductive codes were derived from the argumentation for preferred insulins obtained from literature and a priori expert discussions and inductive codes from the focus groups itself. The coding focussed on identifying reasons for prescribing of the preferred insulins. Those identified perspectives were subsequently classified into the categories of argumentation provided by the model of Cabana et al. (Fig. 1) [25]. This model distinguishes three domains of behaviour change: knowledge, attitudes and external barriers, which are further subdivided into categories. Barriers to guideline adherence related to knowledge can be classified into lack of familiarity with the guideline and lack of awareness. The domain of attitudes consists of lack of agreement (with specific guidelines or guidelines in general), lack of outcome expectancy, lack of self-efficacy and lack of motivation/inertia of previous practice. Both domains, together with external barriers related to patient, guideline and environmental factors define behaviour.

Two out of four transcripts were independently coded by a second researcher (MvdB), using the codebook from the first coder (MD). Any disagreements were solved by discussion between both coders and a third researcher (LvD). The classification of codes into the model of Cabana et al. was discussed within the research team.

## Results

We conducted four focus group discussions. In the two PTAMs, eleven general practitioners, six pharmacists, two nurse practitioners and one practice nurse participated. The two open enrolment groups consisted of eleven practice nurses and four diabetes nurses. The exact number of different professionals per focus group meeting can be found in Table 1.

**Table 1:** Number of participating healthcare professionals in the different focus group discussions

	PTAM 1	PTAM 2	Open enrolment 1	Open enrolment 2	*Total*
General practitioner	5	6			*11*
Pharmacist	3	3			*6*
Nurse practitioner		2			*2*
Practice nurse		1	6	5	*12*
Diabetes nurse			2	2	*4*
*Total*	*8*	*12*	*8*	*7*	*35*

In most practices, the nurses had the most prominent role in initiating insulins, although the general practitioner held the final responsibility. In daily practice, most nurses operated largely independent of the general practitioner, initiating treatments by themselves, including the practice nurses without formal prescriptive authority. Since pharmacists do not initiate insulins themselves, the preferred choices of insulins below refer to general practitioners and nurses only. The perspectives of pharmacists are however included in the analysis of reasons for guideline adherence and non-adherence.

### Choice of insulin

Healthcare professionals were familiar with the five different intermediate or long-acting insulins (NPH insulin, insulin detemir, insulin glargine 100 U/ml, insulin glargine 300 U/ml and insulin degludec) and appreciated their availability to customize the therapy for individual patients. However, almost all healthcare professionals had one preferred insulin they most often initiated and had most experience with.

But it is often a-a-a prescribing preference, you start with something, gain experience with it and then run with it

General practitioner, #1

But I am glad that there are opportunities to switch. And it is also a little customization that you’re providing

Practice nurse, #3

A substantial number of participating healthcare professionals was guideline-adherent, i.e. preferred NPH insulin as first choice in most situations and did not regularly prescribe newer insulins. Positive experience and following the guideline were the most common reasons for the initiation of guideline-recommended insulins. Also, the lower costs and adequate efficacy were mentioned. In a minority of situations, guideline-adherence was prompted by the general practitioner who required the nurse to prescribe guideline-recommended insulins despite her^1^ own preference for other insulins.

### Reasons for guideline non-adherence

During the focus group discussions, two different situations of guideline non-adherence were discussed: the prescription of other insulins than NPH insulin (i.e. insulin glargine 100 U/ml, insulin glargine 300 U/ml, insulin detemir or insulin degludec) to insulin-naïve patients and the prescription of newer insulins (insulin glargine 300 U/ml and insulin degludec), regardless of the former use of other insulins. Some overlap in both situations exists, since the initiation of newer insulins to insulin-naïve patients automatically applies to both situations.

Almost half of the participants preferred the initiation of other insulins than NPH insulin to insulin-naïve patients. Insulin glargine 100 U/ml was the most popular alternative. Although most healthcare professionals did not regularly prescribe newer insulins, almost all had some experience with newer insulins. In most of the cases, newer insulins were prescribed to patients who were already using insulin, but had to switch to another insulin. In addition, a few healthcare professionals preferred the newer insulin degludec as first-choice for all their insulin-naïve patients. Others sometimes initiated newer insulins to insulin-naïve patients because of a specific situation requiring a deviation from their normally preferred insulin.

I think to myself, wait a minute, I’ve done this before [the initiation of new insulins]. I prescribed someone their first Tresiba [insulin degludec], but what was the reason for this? Because the FlexTouch in particular is quite a pleasant device. And it involved someone with a hand disability […]. And then with that the FlexTouch turned out to be an ideal device. So basically I prescribed Tresiba out of practical considerations

Diabetes nurse, #2

In Table 2, all argumentation for the prescription of non-recommended insulins are classified according to the model of Cabana et al. and assigned to both situations of guideline-non-adherence, i.e. initiation of other insulins than NPH insulin to insulin-naïve patients and initiation of newer insulins, regardless of the former use of other insulins. The majority of argumentation applied to both situations. Most of the mentioned reasons were in the domain of attitudes, and especially related to a lack of agreement with guideline recommendations. No barriers in the domains of knowledge were identified. We did discover one new perspective in the domain of attitudes, namely lack of uniformity in policy, which refers to healthcare professionals in the same practice with opposing preferences.

**Table 2:** Argumentation for non-recommended insulins, classified according to Cabana et al. [25].

	Specific argumentation	Initiation of other insulins than NPH insulin to insulin-naïve patients	Initiation of newer insulins
*Knowledge*			
Lack of familiarity			
Lack of awareness (of guideline)			
*Attitudes*			
Lack of agreement	Flexibility in time	X	X
	Hypoglycemia	X	X
	Release profile	X	X
	Future-proof	X	X
	Uniformity device	X	X
	Body weight	X	
	Flexibility injection site	X	
	Injection volume		X
Lack of outcome expectancy	Efficacy	X	X
Lack of self-efficacy			
Lack of motivation/inertion of previous practice	Image/marketing	X	X
	Experience	X	X
Lack of uniformity in policy^a^	Opposing views in the same practice	X	X
*External barriers*			
Patient factors	Inability to resuspend	X	
	Ease of use		X
Guideline factors	Contradictory guidelines		X
Environmental factors	Continuation of prescriptions from other prescribers		X

^a^Newly identified category, not described in the model of Cabana

#### Knowledge

According to Cabana et al., barriers in the domain of knowledge refer to the lack of familiarity with or awareness of the guideline [25]. No such barriers were identified during the focus group discussions.

#### Attitude

Perspectives concerning attitude were most frequent, with lack of agreement as the most prominent category.

##### Lack of agreement

Most perspectives concerning lack of agreement did not reflect disagreement with the guideline recommendations in general, but were about minor advantages which led to better applicability of non-recommended insulins to specific patients in the participants’ view. Healthcare professionals preferred those insulins because of the flexibility, both in injection sites (only mentioned for insulins other than NPH insulin) and time. The flexibility in time was especially mentioned as an advantage for people who make long-distance flights, go on holiday, prefer to sleep in (for example during the weekend) or depend on caregivers for the administration of insulin. During the focus group discussions, this argumentation was put into perspective by some healthcare professionals, stating that the advantage of flexibility only applied to a minority of patients and should not justify the massive use of non-recommended insulins.

Anyway, not everyone wants to sleep in on a Saturday or Sunday and not everyone likes travelling. So you know, I think that’s also a reason to choose a cheaper variant, simply because many people in the Netherlands have and will develop diabetes and will require insulin at some point

Practice nurse, #3

Another perspective concerned the uniformity of devices. For patients combining an intermediate or long-acting insulin with a short-acting insulin, healthcare professionals preferred uniformity in injection devices to enhance the ease of use. In those situations, the type of injection device was more leading than the type of insulin. Healthcare professionals also sometimes chose for a non-recommended insulin taking the future into account. They argued it was better to start with an insulin that would be sufficient for the next years, especially for younger patients. Others opposed this reasoning and stated it was better to start with a cheaper insulin and switch only if necessary, taking into account the higher costs of non-recommended insulins.

Yes, and that's why I think it's somewhat remarkable that practice nurse X just said “I prefer to start with this [insulin degludec], because then I might be able to continue it for a long time”. Meanwhile I'm thinking, you don't know what will be sufficient for the patient. So if you are going to do that [prescribe newer insulins] in advance, you are already going to bet on a very expensive one, while a cheaper one may be sufficient

Practice nurse, #2

Other reasons for preferring non-recommended insulins were related to the release profile which gives a longer time-in-range for patients, making them feel better. Finally, the lower injection volume of insulin glargine 300 U/ml compared to insulin glargine 100 U/ml was mentioned as a reason for the prescription of this newer insulin.

Some healthcare professionals disagreed with the evidence the guideline referred to. According to the guideline, the differences between insulins in effects on hypoglycemia and body weight are marginal and therefore there is no reason to prescribe more expensive insulins [11]. Lower risk of hypoglycemia and less gain of body weight were however used as justification to prescribe other insulins. This argumentation was challenged by others, claiming that the fear of hypoglycemia, both by healthcare professionals and patients, was probably more relevant than the actual risk of hypoglycemia.

But I doubt if it [choosing glargine 100 U/ml instead of NPH insulin because of the risk of hypoglycemia] is because of the fear rather than the actual risk of nocturnal hypoglycemia

General practitioner, #1

##### Lack of outcome expectancy

One perspective concerning the lack of outcome expectancy was identified. Some healthcare professionals preferred non-recommended insulins for poorly controlled T2DM patients, because they believed guideline-recommended insulins had a lower glucose-lowering potential than other insulins. For example, one general practitioner stated she usually prescribed NPH insulin, but chose another insulin if glucose levels were extremely high. She expected other insulins to have a more profound effect on glucose levels.

##### Lack of motivation/inertion of previous practice

Some healthcare professionals chose non-recommended insulins because they had positive experiences (apart from glucose control, which is categorized as ‘outcome expectancy’) after prescribing them. They also pointed out the positive image of 'innovative' insulins. Other healthcare professionals argued that image is mostly constructed by marketing of pharmaceutical industries and mentioned the difficulties of distinguishing real advantages of newer insulins from marketing activities.

But I believe Insulatard [NPH insulin] has a somewhat pompous image. So sometimes you have a relatively young patient and you think, should I choose another one [insulin]? But I think that's more the result of marketing than the actual effect of the medicine

Diabetes nurse, #2

##### Lack of uniformity in policy

One additional category in the domain of attitudes was discussed, referring to a lack of uniformity in policy regarding the prescription of insulins. In some practices, the general practitioner and nurse did not have the same insulin preference, but were not aware of this difference. For example, one general practitioner thought she followed the guideline, prescribing NPH insulin to her patients. But when her actual prescription pattern was analysed, she discovered that most prescriptions were for other insulins. This was due to the preference of the practice nurse, whose prescriptions for non-recommended insulins were authorized by the general practitioner. On the other hand, some nurses stated they wanted to prescribe newer insulins to their patients, but were not allowed to do so, because the general practitioner stimulated them to adhere to the guideline.

Ahh, I prefer NPH insulin. But I checked my actual prescriptions, and then I saw something else. Ahh… the practice nurse, she’d choose Lantus [insulin glargine 100 U/ml] every time

General practitioner, #1

#### External barriers

##### Patient factors

In some situations, patients’ abilities restricted the use of guideline-recommended insulins. For example, patients using NPH insulin need to resuspend the insulin before administration. According to the healthcare professionals, not all patients are capable to do this, thus requiring another insulin. In the same domain, the ease of use of the device was mentioned as reason for the prescription of newer insulins. For example, dysfunctional hand function could require a switch to a non-recommended insulin with a better device applicability.

##### Guideline factors

One guideline-related factor was identified, concerning the prescription of insulin glargine 300 U/ml, namely the presence of contradictory guidelines. A guideline specifically aimed at diabetes nurses gave other recommendations about switching to insulin glargine 300 U/ml (at 40 or 80 units) than the guideline aimed at general practices, which led to confusion.

##### Environmental factors

As environmental factor, the continuation of prescriptions from former prescribers was pointed out. In most cases, this referred to secondary care providers initiating the use of newer insulins. Most healthcare professionals were familiar with internists and/or diabetes nurses from hospitals who initiated newer insulins to their patients, thereby stimulating primary care practitioners to iterate prescriptions for newer insulins. Also, the continuation of insulin prescriptions from other general practitioners for newly registered patients was mentioned.

## Discussion

Although Dutch general practitioners are generally guideline-adherent, the prescription of insulins is often not in line with current treatment recommendations. The present study showed that this non-adherence is largely driven by the lack of agreement with the guideline recommendations, as well as other attitudes of prescribers. A few barriers related to environmental factors, namely patients’ abilities, contradictory guidelines and continuation of prescriptions from other healthcare professionals, were discussed in relation to guideline non-adherence. No factors concerning the knowledge of guideline recommendations were identified.

Our study described two situations of guideline non-adherence: the prescription of other insulins than NPH insulin to insulin-naïve patients and the prescription of newer insulins to all patients. Due to the similarity in argumentation, both situations were analysed and described simultaneously. There are however some differences, especially in the moments when guideline non-adherence occurs. The Dutch guideline T2DM advises NPH insulin as the preferred insulin for all new patients, but provides some room to switch prevalent users of NPH insulin to insulin glargine 100 U/ml or insulin detemir. In contrast, newer insulins (insulin glargine 300 U/ml and insulin degludec) are discouraged for all patients, including prevalent users of insulin [11]. Participants in our study prescribed newer insulins most often to prevalent users, who – according to the healthcare professionals—needed to switch their insulin. Although less frequent, newer insulins were also prescribed to insulin-naïve patients.

The prescription of non-recommended insulins was mostly related to perspectives in the domain of attitudes, which is in accordance with previous studies towards guideline non-adherence in different therapeutical areas in the Netherlands [6, 34]. Most argumentation identified in our study indicated different perspectives on the efficacy (glucose-lowering potential), safety (hypoglycemia, body weight) and applicability (flexibility in injection time and site, applicability of device) of the insulins to patients. This indicates that guideline-non adherence to insulin recommendations is mostly intentional and a deliberate decision of healthcare professionals, which is in line with the results of other studies towards guideline non-adherence [6, 34, 35]. However, the validity of argumentation in the domains of attitudes can be argued – which also occurred during the focus group discussion -, albeit on different levels. First, some perspectives identified in this study can be challenged with the current evidence. For example, the guideline committee that developed the Dutch primary care guideline on T2DM concluded after thorough review of the literature that no clinically relevant differences in hypoglycemia risk exists between intermediate- and long-acting insulins [11]. Nevertheless, the lower risk of hypoglycemia was frequently used as justification by participants in our study to prescribe non-recommended insulins. Reasons like these led often to discussions between the participants in the focus groups, indicating that contrasting interpretation of evidence is indeed an important factor that explains the differences between prescribers in the prescription of insulins. Second, some argumentation (for example flexibility in injection site) do not refer to discussions about evidence, but to the question whether these ‘customization’ perspectives justify the use of more expensive insulins by large groups of patients. In general, guidelines are developed based on population advantages, taking into account the long term outcomes on population level. In daily practice, decisions might be more influenced by other considerations, like short term outcomes on patient level, and less on cost-efficacy on population level [36–38]. Both views can be contradictory if a non-recommended treatment would account for minor advantages for patients at higher costs. Finally, marketing and image of newer insulins were mentioned as important factors in the attitudes towards insulins. Most healthcare professionals were aware of this mechanism, realising that the positive and innovative image of newer insulins was probably mostly constructed by marketing and therefore no valid reason for the prescription of newer insulins. Still, the innovative image did account for the prescription of non-recommended insulins.

We did identify one new category of attitude-related barriers, namely the lack of uniformity in policy. This barrier reflected opposing views in the same practice, with healthcare professionals not being aware of each other preferences. This barrier could lead both to guideline-adherence, in the case one healthcare professional was stimulating the other to prescribe guideline-recommended insulins, and (unintentional) non-adherence, in the case healthcare professionals were not aware of each other’s preference for non-recommended insulins. This newly identified barrier in addition to the model of Cabana et al. most probably reflects the increasing complexity and number of different healthcare professionals in primary care since the development of the model of Cabana in 1999 [28, 31] and points out the importance of good communication and coordination of policy between healthcare professionals.

The lack of knowledge-related barriers for guideline adherence found in our study was not surprising, because of the long history of using clinical guidelines and the prominent role of current guidelines in the post-graduate education of healthcare professionals in primary care in the Netherlands [34]. In addition, T2DM is a frequent condition in primary care [39] and healthcare professionals are well educated about this disease. It can however not be excluded that knowledge-related barriers were overlooked, because healthcare professionals being more familiar with the guideline and treatment of T2DM were more likely to sign up for the focus group discussions. Some external barriers to follow the guideline were identified. The first external barrier reflected barriers at patient level, referring to physical limitations that prevented the use of specific insulins or devices. Second, one guideline-factor was identified, namely the availability of different recommendations about the number of units that require switching from insulin glargine 100 U/ml to insulin glargine 300 U/ml. Third, as environmental barrier, the continuation of prescriptions from other prescribers were mentioned. Although these perspectives were mentioned less often than the barriers in the domain of attitude, these external barriers and explicitly the role of the secondary care should not be marginalized. New medicines initiated by secondary care providers are often subsequently iterated in general practice. Due to this mechanism, primary care providers will become familiar with new medicines, which can lead to adoption of these medicines by the primary care provider herself [40].

The main strength of our study is the use of focus groups with different professionals. Since we included all healthcare professionals, irrespective of their preferred insulins, this resulted in a balanced overview on the preferences and perspectives of the prescription of insulins. In addition, the use of the existing framework to classify barriers to guideline adherence allowed for a thorough evaluation of argumentation. There are also some limitations. The qualitative study design is by definition a possible source of bias, as the interpretation of argumentation and the classification into domains and categories can be subjective. By using two coders and the verification of the coding and classification by a third researcher, we minimised this risk. In addition, since four focus group discussions were budgeted, we did not formally went on with organising until data saturation was reached. However, because few new perspectives were identified during the last focus group and the views of a large number of 35 healthcare professionals were included, we presume data saturation and a complete overview on the topic. Furthermore, selection bias might have occurred in this study, since healthcare professionals interested in the dynamics between guideline-recommendations and actual prescription behaviour were probably more likely to sign up for the focus group discussions. In addition, the use of PTAMs as focus groups might also have limited the range of perspectives found in our study. PTAMs are organised to coordinate and align prescription behaviour. Likely, beliefs and perspectives of healthcare professionals participating in the same PTAM are more uniform than from a random population of healthcare professionals. To obtain a broader view, we additionally organised two focus group discussions for nurses with open enrolment.

The results of our study can be used to develop interventions directed at healthcare professionals in primary care to stimulate rational prescribing of insulins. The prominence of barriers in the domain of attitudes suggests that interventions to stimulate better prescription behaviour should be directed to the views and perspectives of healthcare professionals on insulins, rather than on external barriers and knowledge of the guideline. Most perspectives did not reflect disagreement with the guideline recommendations in general, but were about minor advantages which led to better applicability of other insulins to specific patients in the participants’ view. The finding that healthcare professionals in the focus group discussions regularly challenged each other’s argumentation for non-recommended insulins indicates that there is indeed opportunity for improvement and points out the importance of good and regular communication. Therefore, thorough explanation of treatment recommendations in guidelines, including the description of clinically relevant differences and cost-efficacy is warranted and could stimulate qualitative and cost-effective prescription of insulins.

## Conclusions

This study shed light on the reasons why Dutch primary care practitioners often prefer non-recommended insulins. Lack of agreement with the guideline recommendations and different interpretation of evidence are the most prominent reasons for the prescription of non-recommended insulins. These insights can be used when developing interventions directed at healthcare professionals to stimulate the qualitative and cost-efficient prescription of insulins in primary care.

## Data Availability

The datasets generated and/or analysed during the current study are not publicly available, due to privacy reasons.

## Abbreviations

IRUM: Institute for the Rational Use of Medicine
T2DM: Type 2 Diabetes Mellitus
NHG: Dutch College of General Practitioners
PTAM: PharmacoTherapy Audit Meetings
NPH insulin: Neutral Protamine Hagedorn Insulin

## Footnotes

1 Due to privacy reasons, all healthcare professionals are referred to as 'her', irrespective of sex.
